# 

**Published:** 1881-01

**Authors:** 


### Books and Pamphlets Received During Dec., 1880.

A Treatise on Fractures and Dislocations, By F. H. Hamilton, M.D., LL.D. H. C. Lea's Son & Co., Philadelphia; 1880.

A Treatise on Diphtheria. By A. Jacobi, M.D. Wm. Wood & Co., N. Y.; 1880.

A Practical Treatise on Surgical Diagnosis. By A. L. Ranney, M. D. Wm. Wood & Co., New York; 1880.

The Compend of Anatomy. By J. B. Roberts, M.D. Philadelphia; 1881.

Ophthalmic and Otic Memoranda. By D. B. St. John Roosa, M.D., and Edw. T. Ely, M.D. New York: Wm. Wood & Co.; 1880.

Cutaneous and Venereal Memoranda. By H. G. Pifford, M.D., and Geo. H. Fox, M.D. New York: Wm. Wood & Co.; 1880.

Diet for the Sick. By Jno. Holland, M.D.
An excellent little treatise. Containing much valuable information not generally known.

Transactions American Otological Society, 13th Annual Session. Vol. 2, part 4.

The "Abdominal Method" of Singing and Breathing as a Cause of "Female Weakness." By Clifton E. Wing, M. D., Boston.

Pathology and Treatment of Epulis. By N. Senn, M.D., Milwaukee.

Higher Education of Medical Men and its Influence on the Profession and Public. By F. D. Lente, A.M., M.D.

On the Mangement of Infantile Eczema. By L. D. Bulkley, M.D.

Causation of Idiocy. I. N. Kerlin, M.D.

Annual Report of the Supervising Surgeon-General of the Marine Hospital Service of the United States, for the year 1880.

Pneumo-Dynamics. By G. W. Garland, M.D.

Acts of the Legislature of Louisiana in Regard to Quarantine. By J. Jones, M.D.

Atlas of Histology. By E. Klein, M.D., and H. E. Noble Smith, M.D. Part XI. Philadelphia; 1880. J. B. Lippincott & Co.

---

THE USE OF THE ACTUAL CAUTERY IN ULCERATION OF THE CORNEA. By Dr. Fuchs, (Vienna.)—The application of the actual cautery in cases of ulceration of the cornea was, so far as Dr. Fuchs knew, just adopted by Martinache, of San Francisco, and Gayet, of Lyons. At the meeting of the German Ophthalmological Society, in 1870, Prof. Sattler mentioned the success which had attended it; and Dr. Fuchs had since employed it in appropriate cases in Prof. Arlt's clinic, with encouraging results. The instrument used by him consisted of a ball of the size of a large pea, with an arm like that used by dentists for the destruction of the dental pulp. It was easily heated red in any good gas flame, and was best applied when the iron was beginning to become black. He had used it in abscesses of the cornea and in uleus rodens. The abscesses were partly traumatic and partly spontaneous ; some were the result of smallpox. The application was not followed by any serious reaction. He regarded the action of the cautery as that of a powerful caustic, destroying the suppurating parts and the infectious germs contained in them. Its great advantage consisted in its strict limitation to the affected part. Dr. Fuchs believed Paquelin's cautery, or the galvano-caustic apparatus, liable to become too hot, while the point of the latter was too large for application to the cornea.—*Extract from the British Med. Journal's Report of the Forty-eighth Annual Meeting of the British Medical Association.*

### SOCIETY MEETINGS.

Chicago Medical Society—Mondays, Jan. 3 and 17.
West Chicago Medical Society—Mondays, Jan. 10 and 24.
Biological Society—Wednesday, Jan. 5.

### CLINICS.

MONDAY.
Eye and Ear Infirmary—2 p. m., Ophthalmological, by Prof. Holmes; 3 p. m., Otological, by Prof. Jones.
Mercy Hospital—2 p. m., Surgical, by Prof. Andrews.
Rush Medical College—2 p. m., Dermatological and Venereal, by Prof. Hyde.
Woman's Medical College—2 p. m., Dermatological and Venereal, by Prof. Maynard; 3 p. m., Diseases of the Chest, Prof. Ingals.

TUESDAY.
Cook County Hospital—2 to 4 p. m., Medical and Surgical Clinics.
Mercy Hospital—2 p. m., Medical, by Prof. Quine.

WEDNESDAY.
Chicago Medical College—2 p. m., Eye and Ear, by Prof. Jones.
Rush Medical College—2 p. m., Medical, by Dr. Bridge; 3 p. m., Ophthalmological and Otological, by Prof. Holmes; 3:30 to 4:30 p. m., Diseases of the Chest, by Dr. E. Fletcher Ingals.

THURSDAY.
Chicago Medical College—2 p. m., Gynæcological, by Prof. Jenks.
Rush Medical College—2 p. m., Diseases of Children, by Dr. Knox; 3 p. m., Diseases of the Nervous System, by Prof. Lyman.
Eye and Ear Infirmary—2 p. m., Ophthalmological, by Dr. Hotz.
Woman's Medical College—3 p. m., Surgical, by Prof. Owens.

FRIDAY.
Cook County Hospital—2 to 4 p. m., Medical and Surgical Clinics.
Mercy Hospital—2 p. m., Medical, by Prof. Davis.

SATURDAY.
Rush Medical College—2 p. m., Surgical, by Prof. Gunn; 3 p. m., Orthopœdic, by Prof. Owens.
Chicago Medical College—2 p. m., Surgical, by Prof. Isham; 3 p. m., Neurological, by Prof. Jewell.
Woman's Medical College—11 a. m., Ophthalmological, by Prof. Montgomery; 2 p. m., Gynæcological, by Prof. Fitch.

Daily Clinics, from 2 to 4 p. m., at the Central Free Dispensary, and at the South Side Dispensary.

